# Determination of Cortisol and Dehydroepiandrosterone Levels in Saliva for Screening of Periodontitis in Older Japanese Adults

**DOI:** 10.1155/2009/280737

**Published:** 2010-02-01

**Authors:** Toshihiro Ansai, Inho Soh, Aiko Ishisaka, Akihiro Yoshida, Shuji Awano, Tomoko Hamasaki, Kazuo Sonoki, Yutaka Takata, Tadamichi Takehara

**Affiliations:** ^1^Division of Community Oral Health Science, Department of Health Promotion, Kyushu Dental College, Kitakyushu 803-8580, Japan; ^2^Department of Nutrition, Faculty of Home Economics, Kyushu Women's University, Kitakyushu 807-8586, Japan; ^3^Division of General Internal Medicine, Department of Health Promotion, Kyushu Dental College, Kitakyushu 803-8580, Japan

## Abstract

*Background*. Recent reports have found a positive relationship between periodontitis and the hormones cortisol and dehydroepiandrosterone (DHEA). We investigated the associations between those levels and periodontitis in never-smokers and smokers of elderly subjects. *Subjects and Methods*. Cortisol and DHEA levels in saliva were determined in 171 subjects (85 males, 86 females), with clinical examinations including probing depth (PD) and clinical attachment loss (CAL) also performed. *Results*. Smoking had effects on cortisol and DHEA levels, and those were significantly associated with severe PD and CAL in never-smokers. According to ROC analysis, the cutoff values of cortisol and DHEA to obtain the optimal sensitivity and specificity for detecting severe periodontitis were 2.06 ng/mL and 60.24 pg/mL, respectively, for PD, and 2.12 ng/mL and 61.78 pg/mL, respectively, for CAL. *Conclusions*. Assessment of hormone levels may be a useful screening method for periodontitis, though limited to never-smokers.

## 1. Introduction

A number of investigators have proposed an association between periodontitis and psychosocial stress and the majority of studies in a systematic review [1] found a positive relationship between stress/psychological factors and periodontal disease. However, the relationship between periodontitis and stress-related hormones in saliva is poorly understood. In general, the stress system consists of brain elements, of which the main components are the corticotropin-releasing hormone (CRH) and locus ceruleus-norepinephrine/autonomic systems, as well as their peripheral effectors, the hypothalamic-pituitary-adrenal (HPA) axis, and sympatho-adrenomedullary system [2]. A well-known stress-related hormone is cortisol, while its salivary level reliably reflects HPA activity and has long been used in human psychological studies as a biological marker of stress [3]. Further, DHEA is also known as an HPA-related steroid hormone and has a positive correlation with depression severity [4, 5]. However, few reports regarding salivary DHEA have been presented in the dental field.

Smoking is associated with elevated cortisol and DHEA levels, as it increases the levels of adrenocorticotropin hormone (ACTH) [6]. In addition, smoking is also a major risk for periodontitis [7] and considered likely to be a significant mediator in the relationship between stress-related disorders and the HPA axis [8]. However, to date scant attention has been given to smoking regarding the association between those hormones and periodontal status. We hypothesized that measurement of these hormone levels would be useful for screening for periodontitis in patients who never smoked, since smoking is a stronger risk factor for periodontitis. In the present study, we investigated the associations between those levels and periodontitis in never-smokers and smokers. We also assessed cortisol and DHEA as practical candidate biomarkers for screening of periodontal disease by using receiver operating characteristic (ROC) analyses.

## 2. Subjects and Methods

### 2.1. Population and Samples

The subjects in this cross-sectional study were recruited from members of two senior citizen colleges in Kitakyushu City, Japan. The study sample consisted of community-dwelling, independently living elderly people aged 60 years old and older who attended lectures once a week. These colleges are part of the adult educational system supported by the government of Kitakyushu City, which enrolls students as volunteers for a period of one year. The course of study focused not only on health topics but also on other topics of interest to elderly people, such as finance and culture. The study population voluntarily participated in oral and systemic examinations and initially consisted of 231 subjects (116 males, 115 females) residing in Fukuoka Prefecture, Japan. All of the subjects were independent in daily activities, with none hospitalized at the time of the study, as described previously [9]. Exclusion criteria were as follows: (1) individuals who chronically used corticosteroids and/or immune suppressor drugs as well as those with immune suppressor diseases; (2) individuals with missing questionnaires or saliva data; (3) individuals who used antibiotics within the last 6 months, had symptoms of acute illness, or had any apparent oral infections; and (4) individuals with fewer than 3 natural teeth. As a result, we evaluated a total of 171 subjects (85 males, 86 females; mean age 68.4 ± 4.46 years old) in the present study. We also assessed the differences between the 171 subjects who completed the study and the 60 subjects had been excluded earlier and found no significant differences regarding the tested variables. All subjects understood the nature of the research project and provided written informed consent. The study protocol was reviewed and approved by the Ethics Committee of Kyushu Dental College (No. 05022250).

Before beginning the examinations, each subject was asked to respond to a survey conducted by a dental nurse that consisted of questions related to general medical condition, medication usage, lifestyle, oral health behavior, and oral hygiene habits, and each was also questioned verbally to obtain information regarding smoking status (never, past, or current). The subjects were classified as either never-smokers or smokers (i.e., past, current) on the basis of their answers. Furthermore, a method that used face-scale scores was used to evaluate self-rated health status [10]. From those scores, the subjects were divided into three groups based on overall health (moderate, good, and very good).

### 2.2. Biomarker Analyses

Saliva samples were collected from all subjects between 11:00 AM and 1:00 PM to minimize any circadian rhythm effects, after they had refrained from oral intake for at least 2 hours prior to collection. Subjects with removable partial dentures kept them in their mouth during saliva collection. Collection of stimulated whole saliva was performed using sterile tubes. The subjects were first asked to swallow all saliva in the mouth, then chew paraffin for 3 minutes at a constant pace of 60 times per minute, which was monitored with an electric metronome. Collected samples were placed on ice immediately and the salivary flow rate (mL/min) was estimated by measuring the volume of saliva collected in the tube. Thereafter, the saliva samples were frozen at −30°C until further analysis. The concentration of cortisol in saliva (ng/mL) was measured using a salivary cortisol enzyme immunoassay kit (Salimetrics, State College, PA), with a lower sensitivity limit of 0.07 ng/mL, while that of DHEA (pg/mL) was determined using a salivary DHEA enzyme immunoassay kit (Salimetrics, State College, PA), with a lower sensitivity limit of 10 pg/mL.

### 2.3. Clinical Examinations

Periodontal status was evaluated using probing depth (PD), bleeding on probing (BOP), and clinical attachment loss (CAL). Periodontal examinations were conducted at two sites (mesio-buccal, mid-buccal) of all teeth examined, using a standard periodontal probe (Hu-Friedy, Chicago, IL.) that was inserted into the periodontal pocket parallel to the long axis of all teeth fully erupted in the mouth, according to a modified method described in the Third National Health and Nutrition Examination Survey (NHANES III) [11], with some modifications. All periodontal examinations were performed by two dentists. To confirm interexaminer reliability, duplicate examinations were conducted with 10 outpatients who were visiting Kyushu Dental College Hospital. The intraclass correlation coefficients between examiners for assessment of PD and CAL were 0.72 (95% confidence interval (CI) 0.56–0.82) and 0.74 (95% CI 0.60–0.84), respectively, while those for intraexaminer were 0.78 (95% CI 0.66–0.87) and 0.84 (95% CI 0.74–0.90), respectively. Severe periodontitis was defined as maximum PD  ≥5 mm or maximum CAL  ≥6 mm using the mean value of each cutoff point. Further, in order to evaluate extensive periodontitis, we divided the subjects into three categories (none, low, and high) according to the number of teeth with maximum PD  ≥5 mm or CAL  ≥6 mm, as described previously [9]. Thus, for those with PD, the none group included subjects with no teeth with PD  ≥5 mm, while the low group included those with less than 3 teeth with PD  ≥5 mm, and the high group those with 3 or more teeth with PD  ≥5 mm involved. As for CAL, the none group included subjects with no teeth with CAL  ≥6 mm, while the low group included those with less than 3 teeth with CAL  ≥6 mm, and the high group those with 3 or more teeth with CAL  ≥6 mm involved.

### 2.4. Statistical Analysis

In order to assess differences among the groups, a chi-square test was used for categorized variables and a Kruskal-Wallis test or ANOVA for continuous variables. If a normal distribution was not present according to the results of a Kolomogorov-Smirnov test, the former test was used.

Receiver operating characteristics (ROCs) analyses were performed to determine the optimum cutoff values for the concentrations of cortisol and DHEA, which were used to screen for severe periodontal status. Subjects with severe periodontal status were dichotomized into periodontitis-negative (i.e., none = 0) and periodontitis-positive (i.e., low and high = 1), based on the PD and CAL levels. This dichotomy was then used to calculate the sensitivity and specificity for the cutoff values of cortisol and DHEA. ROC curves were plotted for cortisol and DHEA with PD and CAL and the area under the ROC curve (AUC) was calculated for each. The optimum sensitivity and corresponding specificity were determined from the point on the plot that was closest to the top left-hand corner of the axes. All statistical analyses were performed using SPSS 14.0 for Windows. The level of statistical significance was set at 0.05 for all of the analyses.

## 3. Results

Tables 1 and 2 show subject characteristics of the 3 groups divided according to PD and CAL stratified by smoking status, which were used to analyze the relationships of cortisol and DHEA levels in saliva with periodontal health status. For PD in never-smokers, there were significant differences among the groups regarding age, BOP, and self-rated health status (based on face-scale score), while there were also significant differences for the number of teeth, frequency of tooth brushing, and self-rated health status in the smokers group (Table 1). As for CAL there were significant differences regarding sex and BOP in the never-smokers, while there were significant differences between age and self-rated health status in the smokers (Table 2). As shown in Tables 1 and 2, most of the smokers were male (approximately 96%).

Among never-smokers, the median and 25th and 75th percentile values for cortisol were 1.87, 1.42, and 2.89 (ng/mL), respectively, while those for DHEA were 59.79, 32.69, and 89.52 (pg/mL), respectively. In contrast, among smokers, the median and 25th and 75th percentile values for cortisol were 2.18, 1.41, and 3.01 (ng/mL), respectively, while those for DHEA were 46.07, 30.07, and 89.77 (pg/mL), respectively. However, there were no significant differences regarding those hormone levels between smokers and never-smokers. We compared the levels of cortisol and DHEA between the with and without extensive periodontitis groups, which were defined by the number of teeth with PD  ≥5 mm or CAL  ≥6 mm. Table 3 shows the results of that comparison in the never-smokers, while Table 4 shows the results for smokers. As for the levels of both in the never-smokers, significant differences were found among the three categories defined by PD and CAL, and higher hormone levels were found in subjects with severe PD or CAL. However, in smokers, no significant differences were found among the 3 categories defined by PD or CAL. In contrast, in comparisons of salivary flow rate, there were no significant differences among the three categories for both PD and CAL, irrespective of smoking status (Tables 3 and 4).

**Figure 1:**
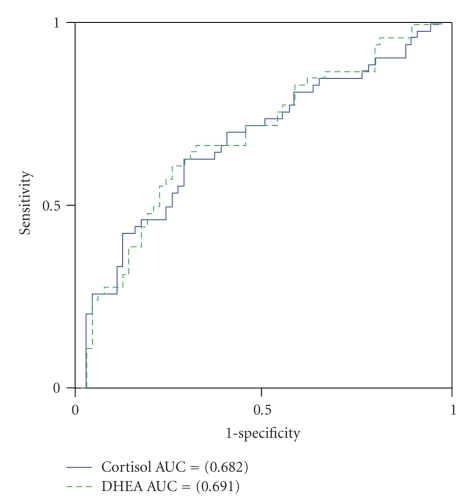
ROC curves used for the cutoff values of cortisol (ng/mL) and DHEA (pg/mL) in saliva to screen PD levels in never-smokers.

**Figure 2:**
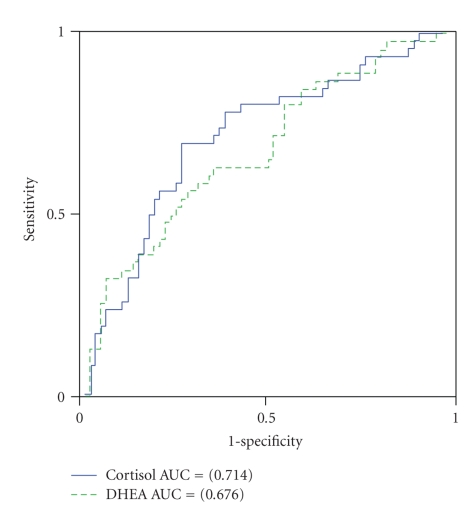
ROC curves used for the cutoff values of cortisol (ng/mL) and DHEA (pg/mL) in saliva to screen CAL levels in never-smokers.

Next we evaluated the usefulness of measuring cortisol and DHEA levels to screen for periodontitis in never-smokers with the above-mentioned associations. For that purpose, we performed ROC analysis to discriminate between false-positive and true-positive diagnoses of severe periodontitis (i.e., low and high) at various cutoff levels. The area under the curve (AUC) for PD was 0.68 (95% CI 0.58–0.78, *P* = .001) and 0.69 (95% CI 0.59–0.79, *P* < .001) for cortisol and DHEA, respectively (Figure 1). For CAL, the AUC was 0.71 (95% CI 0.62–0.81, *P* < .001) and 0.68 (95% CI 0.58–0.78, *P* = .002) for cortisol and DHEA, respectively (Figure 2). As shown in Table 5, the optimal sensitivity and specificity of cortisol for PD were 0.63 and 0.71, respectively, while they were 0.67 and 0.66, respectively, for DHEA, with cutoff values of 2.06 (ng/mL) for cortisol and 60.24 (pg/mL) for DHEA, which indicated a corresponding level in the 55th and 51st percentile, respectively. As shown in Table 6, the optimal sensitivity and specificity of cortisol for CAL were 0.70 and 0.73, respectively, while they were 0.63 and 0.64, respectively, for DHEA, with cutoff values of 2.12 (ng/mL) for cortisol and 61.78 (pg/mL) for DHEA, which indicated a corresponding level in the 56th and 54th percentile, respectively.

## 4. Discussion

In the present cross-sectional study, we investigated cortisol and DHEA levels and periodontal status in elderly subjects and found that levels of the salivary stress-related hormones cortisol and DHEA were useful for screening for periodontitis in subjects who had never smoked. 

The association between periodontitis and stress-related hormones has been largely overlooked, with only two known human studies of the associations between cortisol in saliva and periodontitis reported. One of those was our own survey [9], while the other was a report by Hilgert et al. [12]. In the latter, the authors found a positive association between salivary cortisol and periodontitis, while hypercortisolemia was independently associated with the severity of periodontitis, as defined by CAL (mean CAL  ≥4 mm versus <4 mm), and the extent of periodontitis, as defined by PD (≥26% versus <26% of sites with PD  ≥4 mm) or CAL (≥30% of sites with CAL  ≥5 mm versus <30%). However, smoking status was removed from the final model of logistic regression analysis in that report, while we did not treat smoking as a factor based on stratification of smoking status. In a recent study, treatment of smoking as a confounding factor resulted in a greatly underestimated magnitude of association [13]. 

Smoking is known to be associated with elevated cortisol and DHEA levels. In a recent report that compared cortisol profiles of smokers and nonsmokers over the day, cortisol levels were elevated in everyday life among smokers compared with nonsmokers, and the differences in values were quite substantial, averaging 35% or more on both working and weekend days [6]. In the present study, cortisol levels were higher in smokers than never-smokers, though the differences were not significant (2.18 versus 1.87 ng/mL). Furthermore, salivary levels of cortisol and DHEA were significantly elevated according to the severity of periodontitis, in our never-smoked subjects, whereas no significant association was observed in smokers. One possible explanation for these findings might be that the capability of those hormones to differentiate periodontally affected patients from periodontally healthy individuals is weaker than that of smoking. Conversely, since smoking itself is a stronger risk factor for periodontitis than other known risk factors, evaluation of smoking behavior may be a superior screening method for smokers.

Measurement of biomarkers in saliva has many advantages, as the procedure is stress-free and noninvasive, and allows for frequent and rapid sampling, whereas diurnal rhythm, artificial changes due to food or drinking substances, and blood-contamination are some of the disadvantages. Thus, as described above in the Methods section, the sampling method must be carefully performed. In order to minimize any circadian rhythm effects, we selected the period between 11:00 AM and 1:00 PM for obtaining saliva samples, which has been reported to be stable in regard to daytime hormones levels in nonsmokers [6]. 

A periodontal probe is generally used for periodontitis screening during community-based oral health check-ups, though several problems, including cost, burden on the subject, prevention of infection, and manpower needs, have been pointed out. Our analysis using ROC curves showed acceptable sensitivity and specificity for periodontitis screening with both of the salivary hormones tested in the present study. On the other hand, progress is being made in the development of various screening tests for periodontitis using enzymes [14], cytokines [15], and antimicrobial proteins [16] in saliva. We think that it would be better to combine several tests that reflect the multiple risk factors associated with periodontitis and not depend on results of a single test. On the other hand, it is important to consider the cost-effectiveness when promoting such screening tests for clinical use. A commercial test kit for cortisol costs approximately US$ 250 and can be used 50 subjects, that is, US$ 5.00 per test. However, additional costs must be added when considering the required manpower for the assay and other laboratory charges. In the near future, it is anticipated that screening tests used for a large population will be noninvasive and less burdensome for the subjects, with no requirements of specific devices or instruments, or for individual examinations by an expert as well as reasonable cost-effectiveness performance.

A limitation of the present study is that our subjects were generally in good health and noninstitutionalized. In addition, the percentage of subjects with severely extensive periodontitis among all subjects analyzed was low. Thus, these findings may indicate that the association exists primarily in systemically and orally healthy elderly individuals. Furthermore, it is possible that the periodontal examination method that utilized two sites per tooth in this study, which was based on NHANS III, may underestimate the severity and extent of periodontitis. Additional investigations are necessary to validate and extend the findings using other ages or outpatients. Further, the usefulness of cortisol and DHEA as predictors for periodontitis cannot be determined from our findings, and a longitudinal study is necessary to determine the relationships of those hormones to the progression of periodontitis.

 In summary, we found a significant association between the salivary steroid hormones cortisol and DHEA and periodontitis severity in community-dwelling elderly subjects who had never smoked. These results indicate that these stress-related hormones are useful indicators of the risk for periodontitis, as they showed moderate levels of sensitivity and specificity for periodontitis. Within the study limitations regarding smoking status, the present method of determining cortisol and DHEA levels may include a possibility as screening test for periodontal disease in the near future. Additional approaches to reveal a new type of screening test system for periodontitis as an alternative to the conventional probing method are required.

## Figures and Tables

**Table 1:** Subject characteristics by smoking status based on PD levels.

	Number of teeth with PD ≥5 mm*							
	Never-smokers				Smokers			
Characteristics	None	Low	High	*P* value	None	Low	High	*P* value
Number	59	41	13		28	22	8	
Age in years								
Mean	67.4	69.4	69.5	.043	66.8	69.7	69.6	.065
(SD)	(4.4)	(3.8)	(4.2)		(4.5)	(5.2)	(3.7)	

Number of teeth								
Mean	23.2	22.0	21.5	.565	25.0	20.2	22.5	.030
(SD)	(7.0)	(6.1)	(5.0)		(5.1)	(7.3)	(6.5)	

Site with BOP								
Mean	2.9	4.5	8.0	.003	2.6	4.7	5.5	.149
(SD)	(4.0)	(4.6)	(8.0)		(3.8)	(4.4)	(6.9)	

Sex								
Male	13 (22)	11 (27)	5 (38)	.460	27 (96)	21 (96)	8 (100)	.833

Social class								
Non-workers	54 (92)	33 (80)	12 (92)	.494	27 (97)	20 (91)	8 (100)	.203

Medication	32 (54)	22 (54)	6 (46)	.866	13 (46)	14 (64)	6 (75)	.256

Use of interdental brush								
Yes	32 (54)	21 (51)	7 (54)	.955	12 (43)	10 (46)	5 (63)	.612

Dental visit in past 12 months								
Yes	31 (48)	24 (59)	8 (62)	.759	17 (61)	12 (54)	3 (38)	.506

Frequency of tooth brushing (per day)								
≤1 time	3 (5)	7 (17)	2 (15)	.389	7 (25)	8 (36)	7 (88)	.029
2 times	36 (61)	23 (56)	7 (54)		14 (50)	9 (41)	0 (0)	
≥3 times	20 (34)	11 (27)	4 (31)		7 (25)	5 (23)	1 (13)	

Self-rated health status								
Very good	16 (27)	17 (41)	10 (77)	.016	12 (43)	10 (46)	2 (25)	.001
Good	38 (64)	20 (49)	2 (15)		16 (57)	12 (55)	3 (38)	
Moderate	5 (8)	4 (10)	1 (8)		0 (0)	0 (0)	3 (38)	

*Defined by the number of teeth (no teeth, less than three teeth, three or more teeth) with PD  ≥5 mm. Categorical variables indicate the number of subjects (%). Differences between groups were tested using a chi-square test for categorical variables and ANOVA for continuous variables. PD: probing depth (mm). BOP: bleeding on probing.

**Table 2:** Subject characteristics by smoking status based on CAL levels.

	Number of teeth with PD ≥6 mm*							
	Never-smokers				Smokers			
Characteristics	None	Low	High	*P* value	None	Low	High	*P* value
Number	67	32	14		26	18	14	

Age in years								
Mean	68.2	68.1	70.1	.287	66.5	69.7	69.8	.034
(SD)	(4.5)	(3.9)	(3.8)		(4.1)	(4.7)	(5.4)	

Number of teeth								
Mean	23.4	22.2	19.4	.101	23.9	23.3	20.3	.222
(SD)	(6.5)	(6.7)	(5.2)		(6.6)	(5.1)	(7.5)	

Site with BOP								
Mean	3.0	5.9	5.4	.014	3.8	3.6	4.0	.972
(SD)	(4.2)	(5.5)	(6.3)		(5.1)	(3.4)	(5.3)	

Sex								
Male	12 (18)	9 (28)	8 (57)	.009	25 (96)	17 (94)	14 (100)	.686

Social class								
Non-workers	61 (91)	26 (81)	12 (86)	.614	25 (96)	16 (89)	14 (100)	.601

Medication	38 (56)	15 (49)	7 (50)	.636	13 (50)	11 (61)	6 (64)	.623

Use of interdental brush								
Yes	34 (51)	20 (63)	6 (43)	.392	10 (39)	8 (44)	9 (64)	.289

Dental visit in past 12 months								
Yes	33 (49)	21 (66)	9 (64)	.244	16 (62)	10 (56)	6 (43)	.526

Frequency of tooth brushing (per day)								
≤1 time	6 (9)	3 (9)	3 (21)	.366	6 (23)	10 (56)	6 (43)	.289
2 times	40 (60)	21 (66)	5 (36)		13 (50)	5 (28)	5 (36)	
≥3 times	21 (31)	8 (25)	6 (43)		7 (27)	3 (17)	3 (21)	

Self-rated health status								
Very good	22 (33)	14 (44)	7 (50)	.704	12 (46)	8 (44)	4 (29)	.037
Good	39 (58)	15 (47)	6 (43)		14 (54)	10 (56)	7 (50)	
Moderate	6 (9)	3 (9)	1 (7)		0 (0)	0 (0)	3 (21)	

*Defined by the number of teeth (no teeth, less than three teeth, three or more teeth) with PD  ≥6 mm. Categorical variables indicate the number of subjects (%). Differences between groups were tested using a chi-square test for categorical variables and ANOVA for continuous variables. CAL: clinical attachment level (mm). BOP: bleeding on probing.

**Table 3:** Median values for salivary cortisol and dehydroepiandrosterone in presence or absence of extensive periodontitis in subjects who never smoked, divided by PD and CAL.

	Categories divided by PD*			
	None	Low	High	*P* value^†^
Number	59	41	13	
Cortisol (ng/mL)				
Median	1.68	2.32	3.10	.002
(25th, 75th percentile)	(1.29, 2.40)	(1.58, 3.31)	(2.08, 3.62)	

DHEA (pg/mL)				
Median	47.22	68.99	119.01	<.001
(25th, 75th percentile)	(28.50, 70.29)	(37.49, 97.93)	(79.67, 137.93)	

Salivary flow rate (mL/min)				
Median	0.90	0.97	0.90	.578
(25th, 75th percentile)	(0.67, 1.13)	(0.67, 1.33)	(0.60, 1.52)	

	Categories divided by CAL*			
	None	Low	High	*P* value^†^

Number	67	32	14	
Cortisol (ng/mL)				
Median	1.64	2.49	2.98	<.001
(25th, 75th percentile)	(1.29, 2.39)	(1.75, 3.10)	(1.84, 3.67)	

DHEA (pg/mL)				
Median	54.28	66.60	104.44	.003
(25th, 75th percentile)	(28.50, 76.69)	(41.57, 111.30)	(68.62, 122.41)	

Salivary flow rate (mL/min)				
Median	0.93	1.08	0.75	.092
(25th, 75th percentile)	(0.67, 1.17)	(0.73, 1.38)	(0.31, 1.06)	

*Defined by the number of teeth (no teeth, less than 3 teeth, 3 or more teeth) with PD  ≥5 mm or CAL  ≥6 mm. DHEA: dehydroepiandrosterone. PD: probing depth (mm). CAL: clinical attachment loss (mm). ^†^Kruskal-Wallis test.

**Table 4:** Median values for salivary cortisol and dehydroepiandrosterone in presence or absence of extensive periodontitis in subjects who smoked, divided by PD and CAL.

	Categories divided by PD*			
	None	Low	High	*P* value^†^
Number	28	22	8	
Cortisol (ng/mL)				
Median	2.01	2.13	2.44	.573
(25th, 75th percentile)	(1.41, 3.13)	(1.36, 2.73)	(1.98, 3.13)	

DHEA (pg/mL)				
Median	36.72	48.70	94.75	.173
(25th, 75th percentile)	(28.44, 82.12)	(30.07, 89.77)	(46.67, 121.26)	

Salivary flow rate (mL/min)				
Median	0.87	0.80	0.92	.494
(25th, 75th percentile)	(0.51, 1.26)	(0.29, 1.19)	(0.69, 1.25)	

	Categories divided by CAL*			
	None	Low	High	*P* value^†^

Number	26	18	14	
Cortisol (ng/mL)				
Median	2.01	2.09	2.59	.243
(25th, 75th percentile)	(1.29, 2.58)	(1.42, 3.09)	(1.68, 3.43)	

DHEA (pg/mL)				
Median	45.12	44.30	55.98	.633
(25th, 75th percentile)	(32.99, 85.14)	(19.49, 95.35)	(30.54, 114.47)	

Salivary flow rate (mL/min)				
Median	0.90	0.88	0.59	.079
(25th, 75th percentile)	(0.66, 1.21)	(0.57, 1.56)	(0.29, 1.05)	

*Defined by the number of teeth (no teeth, less than 3 teeth, 3 or more teeth) with PD  ≥5 mm or CAL  ≥6 mm. DHEA: dehydroepiandrosterone. PD: probing depth (mm). CAL: clinical attachment loss (mm). ^†^Kruskal-Wallis test.

**Table 5:** Cutoff values for cortisol and DHEA in saliva for optimal sensitivity and specificity obtained from ROC curve for PD levels in never-smokers.

	Cutoff value	Optimal sensitivity	Optimal specificity
Cortisol (ng/mL)	2.06	0.63	0.71
DHEA (pg/mL)	60.24	0.67	0.66

**Table 6:** Cutoff values for cortisol and DHEA in saliva for optimal sensitivity and specificity obtained from ROC curve for CAL levels in never-smokers.

	Cutoff value	Optimal sensitivity	Optimal specificity
Cortisol (ng/mL)	2.12	0.70	0.73
DHEA (pg/mL)	61.78	0.63	0.64

## References

[1] Peruzzo DC, Benatti BB, Ambrosano GM (2007). A systematic review of stress and psychological factors as possible risk factors for periodontal disease. *Journal of Periodontology*.

[2] Chrousos GP, Gold PW (1992). The concepts of stress and stress system disorders: overview of physical and behavioral homeostasis. *Journal of the American Medical Association*.

[3] Dickerson SS, Kemeny ME (2004). Acute stressors and cortisol responses: a theoretical integration and synthesis of laboratory research. *Psychological Bulletin*.

[4] Michael A, Jenaway A, Paykel ES, Herbert J (2000). Altered salivary dehydroepiandrosterone levels in major depression in adults. *Biological Psychiatry*.

[5] Assies J, Visser I, Nicolson NA (2004). Elevated salivary dehydroepiandrosterone-sulfate but normal cortisol levels in medicated depressed patients: preliminary findings. *Psychiatry Research*.

[6] Steptoe A, Ussher M (2006). Smoking, cortisol and nicotine. *International Journal of Psychophysiology*.

[7] Tomar SL, Asma S (2000). Smoking-attributable periodontitis in the United States: findings from NHANES III. National Health and Nutrition Examination Survey. *Journal of Periodontology*.

[8] Olff M, Meewisse ML, Kleber RJ (2006). Tobacco usage interacts with postdisaster psychopathology on circadian salivary cortisol. *International Journal of Psychophysiology*.

[9] Ishisaka A, Ansai T, Soh I (2007). Association of salivary levels of cortisol and dehydroepiandrosterone with periodontitis in older Japanese adults. *Journal of Periodontology*.

[10] Lorish CD, Maisiak R (1986). The face scale: a brief, nonverbal method for assessing patient mood. *Arthritis and Rheumatism*.

[11] Albandar JM, Brunelle JA, Kingman A (1999). Destructive periodontal disease in adults 30 years of age and older in the United States, 1988–1994. *Journal of Periodontology*.

[12] Hilgert JB, Hugo FN, Bandeira DR, Bozzetti MC (2006). Stress, cortisol, and periodontitis in a population aged 50 years and over. *Journal of Dental Research*.

[13] Hyman JJ, Reid BC (2004). Cigarette smoking, periodontal disease, and chronic obstructive pulmonary disease. *Journal of Periodontology*.

[14] Nomura Y, Tamaki Y, Tanaka T (2006). Screening of periodontitis with salivary enzyme tests. *Journal of Oral Science*.

[15] Frodge BD, Ebersole JL, Kryscio RJ, Thomas MV, Miller CS (2008). Bone remodeling biomarkers of periodontal disease in saliva. *Journal of Periodontology*.

[16] Ito T, Komiya-Ito A, Arataki T (2008). Relationship between antimicrobial protein levels in whole saliva and periodontitis. *Journal of Periodontology*.

